# Diagnosis and Management of Mixed Phenotype Hereditary Transthyretin Amyloidosis: A Case-Based, Canadian Perspective

**DOI:** 10.1016/j.cjco.2025.03.002

**Published:** 2025-03-10

**Authors:** Nowell Fine, Anique Ducharme, Genevieve Matte, Michelle Mezei, Vera Bril, Diego Delgado

**Affiliations:** a Division of Cardiology, Department of Cardiac Sciences, Libin Cardiovascular Institute, Cumming School of Medicine, University of Calgary, Calgary Alberta, Canada; b Department of Medicine, Montreal Heart Institute, Université de Montréal, Montreal, Quebec, Canada; c Division of Neurology, Department of Medicine, Centre hospitalier de l’Université de Montréal (CHUM), Montréal, Quebec, Canada; d Division of Neurology, University of British Columbia, Vancouver, British Columbia, Canada; e Ellen & Martin Prosserman Centre for Neuromuscular Diseases, Toronto General Hospital, University Health Network, University of Toronto, Toronto, Ontario, Canada; f Division of Cardiology, Peter Munk Cardiac Center, Toronto General Hospital, Toronto, Ontario, Canada and Division of Cardiology and Cardiac Transplantation, University of Toronto, Toronto, Ontario, Canada

## Abstract

Hereditary amyloid transthyretin variant (ATTRv) amyloidosis is a rare, life-threatening disease, characterized by the deposition of aggregated transthyretin (TTR) protein in multiple organs and tissues. Diagnosis is often delayed due to its heterogeneity in presentation, which includes a wide range of cardiac and/or neurologic symptoms. Thus, awareness of ATTRv amyloidosis across multiple specialties is needed for its early diagnosis and management. This paper provides a review surrounding the diagnosis and management of mixed phenotype ATTRv amyloidosis, addressed through 3 clinical questions. This paper discusses: (i) the need for patients with ATTRv amyloidosis to be screened for mixed cardiac and neurologic phenotypes through early multidisciplinary referral; (ii) the therapeutic landscape for ATTRv amyloidosis in Canada, with emphasis on the need for prompt therapy selection and initiation, based on multidisciplinary collaboration; and (iii) how disease can be monitored pre- and post-treatment. Case studies are provided to illustrate how the available evidence impacts practice.

## Résumé

La variante héréditaire de l'amyloïde à transthyrétine (ATTRh) est une maladie rare, potentiellement mortelle, caractérisée par le dépôt de protéines de transthyrétine (TTR) agrégées dans de multiples organes et tissus. Le diagnostic est souvent retardé en raison de sa présentation hétérogène, qui inclut un large éventail de symptômes cardiaques et/ou neurologiques. Il est donc nécessaire de sensibiliser les différentes spécialités médicales à l'amylose ATTRh afin de permettre un diagnostic et une prise en charge précoces. Cet article fait le point sur le diagnostic et la prise en charge de l'ATTRh à phénotype mixte, à travers trois questions cliniques. Il aborde les points suivants 1) la nécessité de dépister les phénotypes cardiaques et neurologiques mixtes chez les patients atteints d'ATTRh par une orientation multidisciplinaire précoce; 2) le paysage thérapeutique de l'ATTRh au Canada, en mettant l'accent sur la nécessité d'une sélection et d'une initiation rapides du traitement, basées sur une collaboration multidisciplinaire; et 3) la manière dont la maladie peut être surveillée avant et après le traitement. Des études de cas illustrent l'impact des données disponibles sur la pratique.

**Figure 1:**
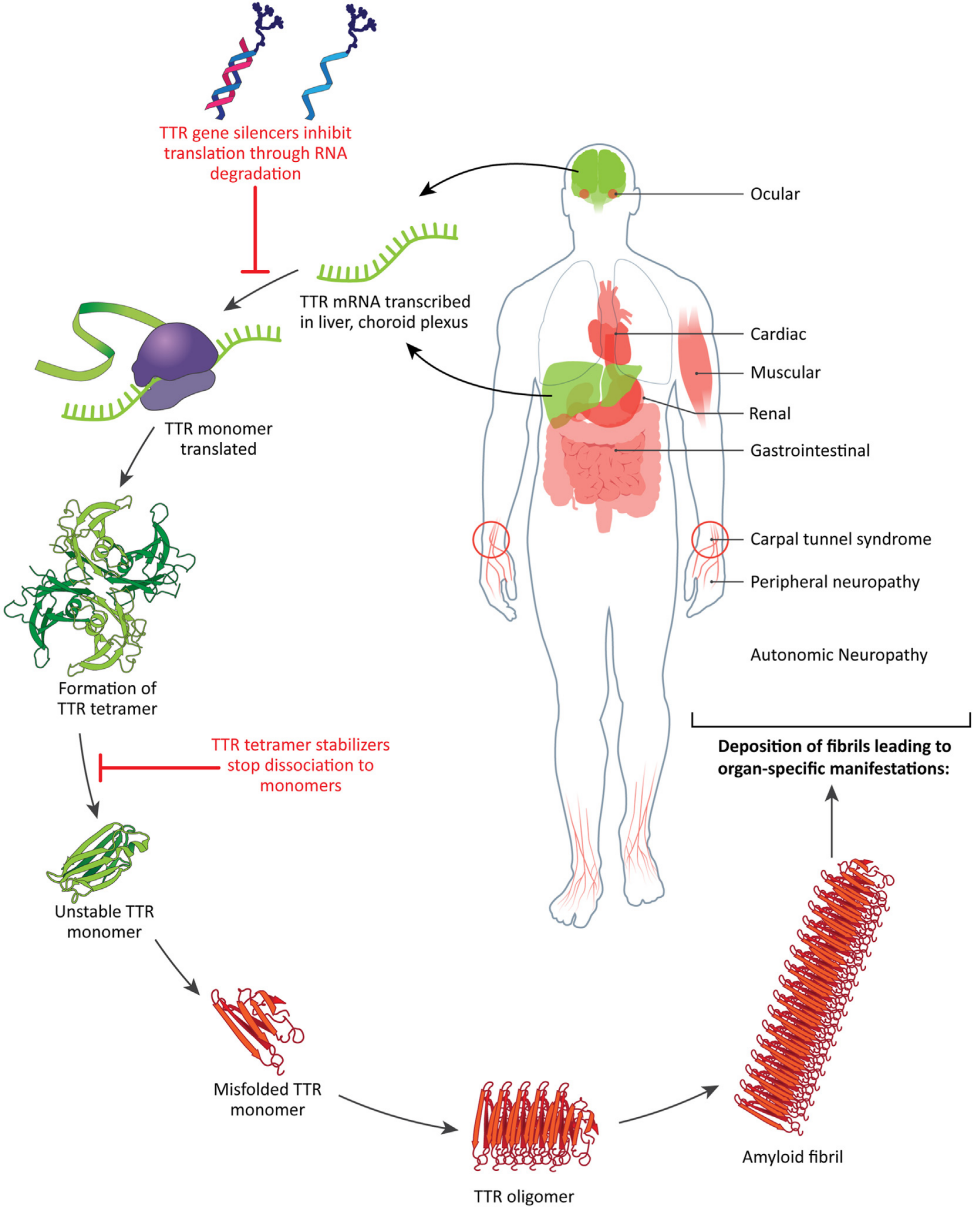
Pathobiology and disease manifestations of amyloid transthyretin variant (ATTRv) amyloidosis. Transthyretin (TTR) is expressed primarily in the liver, with some expression in the choroid plexus, and retinal pigment epithelium. TTR functions as a tetrameric structure made of 2 homodimers, which creates a binding site for thyroid hormone and retinol to facilitate their transport. Variants of the TTR protein can lead to destabilization of the tetramer and dissociation into unstable monomers that can become misfolded. Dissociation is also possible with wild-type TTR. Misfolded TTR monomers can aggregate into TTR oligomers and travel to multiple organs and tissues where they can form amyloid fibrils, causing multisystem dysfunction. Currently, 2 classes of disease-modifying treatment have been developed for ATTRv amyloidosis—TTR gene silencers and TTR tetramer stabilizers—that target different points in the disease pathway to prevent amyloid formation. mRNA, messenger RNA.

Amyloid transthyretin amyloidosis is a debilitating and life-threatening disease caused by the misfolding and aggregation of mutated transthyretin (TTR) protein, which deposits in multiple organs and tissues, leading to progressive damage and dysfunction (Fig. 1). The condition can occur sporadically, with aggregation of wild-type protein (typically presenting as cardiomyopathy in patients aged > 60 years), or it can be associated with mutations in the *TTR* gene, which is inherited in an autosomal dominant manner (hereditary or amyloid TTR variant [ATTRv] amyloidosis).^1^

The clinical presentation of ATTRv amyloidosis is heterogeneous, with some patients having predominantly neurologic or cardiac disease, and others displaying a mixed phenotype of both. Disease presentation can also evolve over time, with data from the longitudinal observational Transthyretin Amyloidosis Outcomes Survey (THAOS) study indicating an increase in the rate of mixed phenotype presentation over the course of the study, from 24% to 34% of all patients with ATTR amyloidosis enrolled.^2^ TTR genotype and ethnicity and/or regional factors play a role in phenotype manifestation, mutation penetrance, disease severity, and age of onset. However, significant heterogeneity in clinical presentation still exists within TTR genotypes and ethnic groups, which further complicates the characterization, and thus the diagnosis and management of this disease.^3^^,^^4^

Hereditary ATTR amyloidosis is considered a rare disease, with a prevalence range of 1-204 cases per million.^5^ Higher rates are reported in endemic populations, such as those in Portugal, Sweden, Brazil, and Japan, where an earlier onset of disease is typically seen (diagnosis between ages 20 and 50 years).^5^ ATTRv amyloidosis in nonendemic regions, such as Canada, is typically diagnosed in individuals aged > 70 years. No studies have assessed the prevalence of this disease in Canada, but estimates of 0.5-1 cases per million people have been suggested, based on extrapolation from rates in other nonendemic countries.^5^^,^^6^ However, regional differences in the prevalence of ATTRv amyloidosis across Canada may exist, and overall, the prevalence is likely to be underestimated due to variability in clinical presentation and reduced diagnostic awareness.^6^^,^^7^ Indeed, diagnosis of ATTRv amyloidosis in nonendemic regions typically takes longer than 3 years, with patients consulting with multiple physicians before a diagnosis is made.^8^ Diagnosis of a mixed phenotype could take an additional 2-4 years after the initial diagnosis, highlighting the importance of early involvement of a multidisciplinary team.^2^

The duration of survival from the time of diagnosis can vary widely, depending on the duration of symptoms prior to diagnosis, TTR genotype, and clinical presentation, with cardiac involvement associated with a poorer prognosis than that for isolated polyneuropathy.^1^ Liver transplantation to prevent the expression of mutant TTR protein was the first intervention to demonstrate improved rates of long-term survival in patients with early-onset ATTR p.V50M (V30A) amyloidosis.^9^ However, this procedure is associated with a high morbidity rate and a shorter duration of benefit in other genotypes.^9^ Novel disease-modifying therapies that target TTR amyloid production via multiple mechanisms largely have replaced liver transplantation as the standard-of-care. In Canada, these include the use of TTR protein stabilizers, which are currently indicated to treat ATTR cardiomyopathy, and the use of *TTR* gene silencers, which are currently indicated to treat ATTRv polyneuropathy. In general, the greatest benefit from these agents is achieved in those with early treatment initiation after symptomatic onset,^10^ which underscores the importance of increasing the early diagnosis of ATTRv amyloidosis through greater awareness of its clinical presentation.

Given the delayed diagnosis of mixed phenotype ATTRv amyloidosis, building greater awareness on the heterogeneous clinical presentation of this disease among general cardiologists and neurologists is important to allow for early therapeutic intervention and preservation of quality of life. Current international consensus-based guidelines provide valuable frameworks for the diagnosis and management of ATTRv amyloidosis, with either predominantly cardiac or neurologic phenotypes^6^^,^^11^, ^12^, ^13^, ^14^; however, they lack specific recommendations for the treatment and monitoring of patients with a mixed phenotype. This paper, directed at both general and specialized cardiologists and neurologists, uses case studies to illustrate the nuances in the diagnosis, treatment, and monitoring of mixed phenotype ATTRv amyloidosis, through a Canadian lens.

## Methodology

A pan-Canadian panel of 3 cardiologists and 3 neurologists with expertise in the management of patients with ATTRv amyloidosis was formed to discuss key challenges in the diagnosis, monitoring, and treatment of ATTRv amyloidosis with mixed phenotype. Via e-mail and video conference communication, panelists determined 3 priority clinical questions to address. These questions were as follows: (1) Which patients should be screened for mixed phenotype ATTRv amyloidosis?; (2) How should patients with ATTRv amyloidosis with mixed phenotype be treated?; and (3) How should patients with mixed phenotype ATTRv amyloidosis be monitored pre- and post-treatment? Authors provided individual oral feedback on each clinical question to guide the literature review and background content. Clinical cases were shared by 2 authors (D.D. and G.M.) to illustrate important considerations in the diagnosis, management, and monitoring of ATTRv amyloidosis. A draft summarizing initial feedback and clinical cases was developed and circulated to all authors for final comments and approval.

## Clinical Question 1: Which Patients Should Be Screened for Mixed Phenotype ATTRv Amyloidosis?

### Background

**Figure 2:**
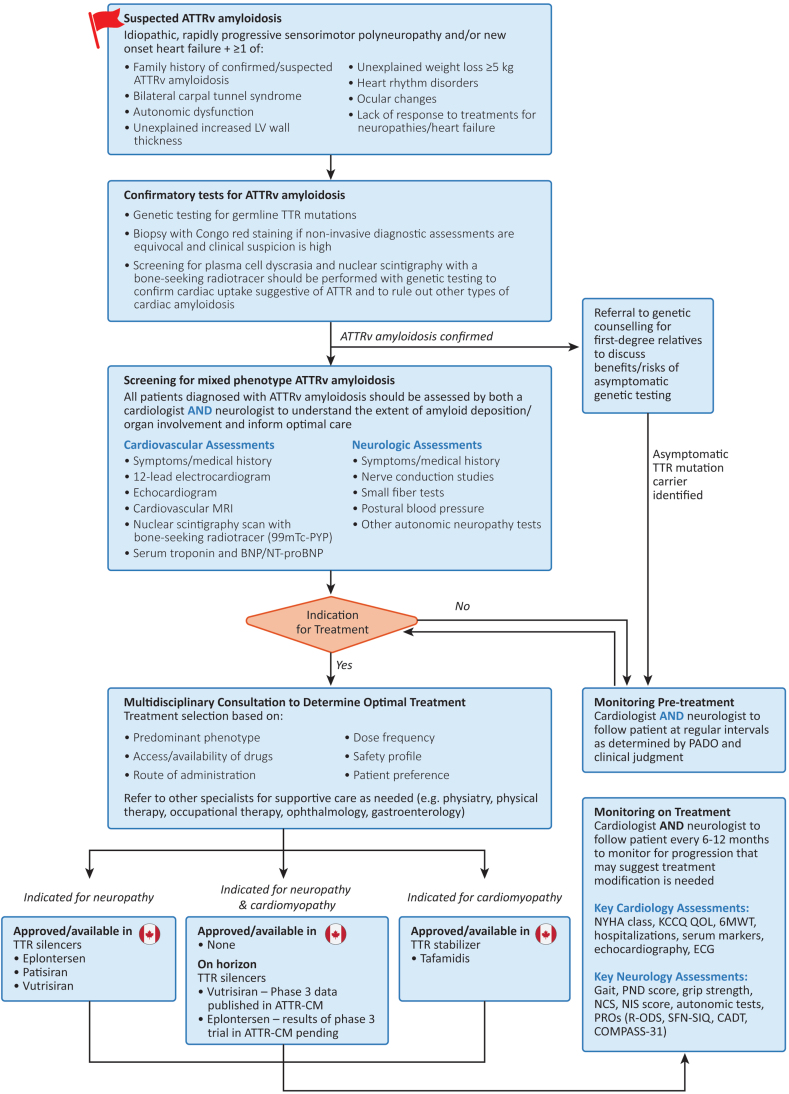
Overview of pathway for diagnosis, treatment, and monitoring in amyloid transthyretin variant (ATTRv) amyloidosis. BNP/NT-proBNP, B-type natriuretic peptide/N-terminal pro B-type natriuretic peptide; CADT, Compound Autonomic Dysfunction Test; CM, cardiomyopathy; COMPASS-31, Composite Autonomic Symptom Score-31; ECG, electrocardiogram; KCCQ QOL, Kansas City Cardiomyopathy Questionnaire Quality of Life; LV, left ventricular; MRI, magnetic resonance imaging; NCS, Nerve Conduction Studies; NIS, Neuropathy Impairment Score; NYHA, New York Heart Association; PADO, predicted age of symptomatic disease onset; PND, polyneuropathy disability scale; PRO, patient-reported outcomes; PYP, pyrophosphate; R-ODS, Rasch-built Overall Disability Scale; SFN-SIQ, Small Fiber Neuropathy-Symptoms Inventory Questionnaire; TTR, transthyretin; 6MWT, 6-minute walk test; 99mTc-PYP, technetium-99 m pyrophosphate.

Before diagnosis, patients with ATTRv amyloidosis may be referred to a neurologist or cardiologist, in cases in which initial investigations and medical history raise suspicion of amyloidosis. The signs and symptoms that should trigger clinicians to suspect ATTRv amyloidosis, and hence initiate a diagnostic workup, are well described (Fig. 2).^6^^,^^11^^,^^13^^,^^15^, ^16^, ^17^ In general, these include a combination of symptoms suggesting sensory or autonomic neuropathy, as well as heart failure and other cardiac symptoms.

In individuals with symptoms of polyneuropathy, diagnosis of ATTRv amyloidosis typically is confirmed with a positive result on genetic testing for germline *TTR* mutations.^6^ For ATTRv cardiomyopathy, screening for plasma cell dyscrasia and nuclear scintigraphy with a bone-seeking radiotracer (eg, a Tc99m-pyrophosphate [PYP] scan) must be utilized together with genetic testing to confirm cardiac uptake suggestive of ATTR and to rule out other types of cardiac amyloidosis.^11^ Although a biopsy to confirm amyloid deposition in the tissue of interest historically has been required for diagnosis, biopsy is now usually reserved for situations in which a high clinical suspicion remains and noninvasive diagnostic assessments are equivocal.^6^^,^^11^ Diagnosis of ATTRv amyloidosis should trigger a suggestion for first-degree relatives to be referred to genetic counseling so that the benefits and risks of genetic testing in asymptomatic individuals can be discussed.^18^

Over 150 TTR variants related to hereditary amyloidosis have been reported.^19^ Although data on genotype distribution in Canada is lacking, in the US, the most common genotypes are p.V142I (V122I), p.T80A (T60A), and p.V50M (V30M), accounting for approximately 45%, 20%, and 6% of ATTRv amyloidosis cases, respectively.^20^ These data are in contrast to data from Europe, South America, and Japan, where the p.V50M (V30M) genotype accounts for over 70% of cases.^4^ The specific TTR variant identified can provide some insight on phenotype expression^14^; however, variability in presentation within genotypes is also present.^2^^,^^21^^,^^22^ For example, the p.V142I genotype, which predominantly affects Americans of African Caribbean descent, historically has been associated with isolated cardiomyopathy with minimal or absent neurologic involvement.^20^ However, higher rates of neurologic involvement in patients with the p.V142I genotype have been reported in studies where neurologic screening was performed, and in general, recent studies have found a significant heterogeneity in phenotype presentation for p.V142I ATTRv amyloidosis.^2^^,^^23^, ^24^, ^25^, ^26^

A mixed phenotype may also present over time, with patients being initially asymptomatic or mildly symptomatic.^2^ Thus, assessment by both a cardiologist and neurologist is important for all patients diagnosed with ATTRv amyloidosis, to best understand the extent of disease and to collaborate on the best treatment and monitoring strategy.

Table 1 outlines recommended investigations to screen for mixed phenotype ATTRv amyloidosis that also are applicable to the diagnostic workup for single system manifestations of the disease.^6^^,^^11^ These tests should be ordered and interpreted by the appropriate specialist. All patients who have confirmed ATTRv cardiomyopathy should be referred to a neurologist for comprehensive neurologic assessment of motor, sensory, and reflex dysfunction and quantitative baseline assessment of large-fiber nerve involvement through nerve conduction studies and electromyography. As disease typically impacts small fibers first, nerve injury may not be identified by nerve conduction studies. In cases in which autonomic and/or somatic small-fiber involvement is unclear based on symptom history and neurologic assessment, tests to confirm small-fiber involvement are needed. This testing may include assessment of intraepidermal nerve-fiber density from a skin-punch biopsy, which can correlate with disease severity and capture abnormalities before symptoms arise.^27^^,^^28^ There are no gold-standard noninvasive small-fiber function tests; however, the combination of multiple tests, depending on local availability, can be used to confirm small-fiber neuropathy (Table 1). Small-fiber neuropathy leading to autonomic dysfunction may be difficult to recognize, as it may be subclinical in early stages, it can overlap with other more common diagnoses (eg, irritable bowel syndrome), and patients may not disclose specific symptoms (eg, erectile dysfunction). Specialized assessment for autonomic neuropathy is not widely available to Canadian clinicians. One exception is postural blood pressure, which is an easy and reliable test to detect orthostatic hypotension that occurs as a result of autonomic nerve dysfunction in approximately half of patients with ATTRv polyneuropathy,^29^ and thus is an important assessment tool.

**Table 1:** Useful assessments and diagnostic tests to screen for mixed phenotype amyloid transthyretin variant (ATTRv) amyloidosis

System involvement	Assessment and/or test	Suggestive of ATTRv involvement
**Cardiac**	**Symptoms; medical history**	• Symptoms of heart failure (eg, dyspnea, fatigue, peripheral edema)
	**Electrocardiogram**	• Normal or low QRS voltage (often discordant with echocardiography findings) • Pseudo-infarct pattern • Conduction blocks (atrioventricular, bundle branch) • Atrial arrhythmia
	**Echocardiography**	• Increased LV wall thickness (≥ 12 mm) with small or normal LV chamber size and increased LA volume (in the absence of pressure overload) • Impaired global longitudinal strain with apical sparing and preserved LVEF • Other co-presenting features may include: o Increased valvular and interatrial septum thickness o Any range of diastolic dysfunction o Small pericardial effusion
	**Cardiac MRI (alternative option to echocardiography)**	• Elevated native T1 relaxation time • Reduced post-contrast T1 relaxation time • Diffuse subendocardial or transmural late gadolinium enhancement • Increased extracellular volume fraction • Abnormal gadolinium kinetics on modified Look-Locker inversion recovery sequence
	**Nuclear scintigraphy – 99mTc-PYP scan**	• Grade 2/3 myocardial uptake (cardiac uptake equal to or greater than bone) • Heart-to-contralateral-lung uptake ratio ≥ 1.5
	**Serum troponin**	• Persistent mild elevation
	**BNP/NTproBNP**	• Persistent high elevation, often disproportionate to the severity of clinical heart failure
**Neurologic**	**Symptoms; medical history; neurologic assessment**	• Peripheral sensorimotor neuropathy (eg, numbness, pain, and/or tingling in feet and hands) • Bilateral carpel tunnel syndrome (often predates other symptoms) • Abnormal gait • Balance disorder • Symptoms suggesting autonomic neuropathy o Gastrointestinal issues (diarrhea or constipation, nausea) o Sweating abnormalities o Sexual dysfunction o Urinary incontinence and/or recurrent UTI o Intolerance to antihypertensive medications (orthostatic hypotension)
	**Nerve conduction studies and electromyography for assessment of large-fiber involvement**∗	• Bilateral carpal tunnel syndrome (often predates other symptoms) • Length-dependent, sensorimotor, axonal peripheral neuropathy (most common) • Motor-predominant neuropathy (mimicking motor neuron disease) • Upper extremity–predominant peripheral neuropathy • Mixed axonal and demyelinating neuropathy with areflexia (mimicking chronic inflammatory demyelinating polyradiculoneuropathy)
	**Skin punch biopsy and other small-fiber tests**†**(If large-fiber tests are negative and clinical evidence of small-fiber involvement is equivocal)**	**Skin punch biopsy:** • Intraepidermal nerve-fiber density below 5th percentile • Axonal swellings • Abnormal nerve fiber orientation • Very fine–caliber axons • Excessive or complex nerve-fiber branching
	**Postural blood pressure or tilt-table test**	• Orthostatic hypotension defined as a ≥ 20 mm Hg decrease in systolic blood pressure or ≥ 10 mm Hg decrease in diastolic blood pressure within 3 minutes of changing from a supine position to standing or—60-degree angle on a tilt-table test
**Musculoskeletal**	**Symptoms; medical history**	• Muscle weakness • Arthropathy • Fatigue • Cachexia/weight loss • Spinal stenosis • Spontaneous biceps tendon rupture
**Gastrointestinal**	**Symptoms; medical history**	• Nausea, constipation, early satiety, abdominal bloating (gastroparesis might be secondary to dysautonomia and/or gastrointestinal involvement) • Elevated liver enzyme levels
**Ocular**	**Symptoms; medical history**	• Vitreous opacities • Glaucoma
**Nephrological**	**Symptoms; medical history**	• Proteinuria • Kidney failure

BNP/NT-proBNP, B-type natriuretic peptide/N-terminal-proBNP; LA, left atrial; LV, left ventricular; LVEF, left ventricular ejection fraction; MRI, magnetic resonance imaging; PYP, pyrophosphate; UTI, urinary tract infection.

∗ Some evidence supports the utility of magnetic resonance neurography (MRN) and peripheral nerve ultrasonography to detect nerve injury in ATTRv amyloidosis.^56^, ^57^, ^58^, ^59^

† No gold standard exists for noninvasive small-fiber tests. Some types of testing that may be used include quantitative sudomotor axon reflex testing, axon-reflex-flare testing, sympathetic skin responses, electrochemical skin conductance, quantitative sensory thresholds, and *in vivo* corneal confocal microscopy.^60^, ^61^, ^62^, ^63^, ^64^, ^65^ Multiple noninvasive tests are required to determine small-fiber involvement.

All patients who have confirmed ATTRv polyneuropathy should be referred to a cardiologist for assessment of cardiac involvement. This assessment includes documenting symptoms and reviewing the patient’s medical history, with particular interest in heart failure symptoms, conductance disorders, and arrhythmias. Electrocardiogram (ECG), echocardiogram or cardiac magnetic resonance imaging (MRI), and serum biomarkers are the first investigations used to assess a potential cardiac manifestation of amyloidosis (Table 1). The sign most suggestive of cardiac involvement is unexplained increased left ventricular (LV) wall thickness. This symptom commonly occurs with preserved left ventricular ejection fraction (LVEF) and discordant ECG results, with the expected high QRS voltage being absent and instead, a pseudo-infarction pattern is seen. In cases in which cardiac MRI is used, late gadolinium enhancement in a diffuse transmural or subendocardial pattern is suggestive of ATTRv.

Nuclear scintigraphy with Tc99m-PYP has a high sensitivity for cardiac involvement in ATTRv amyloidosis and should be ordered regardless of echocardiographic findings in patients with a confirmed ATTR mutation, as positive myocardial uptake can sometimes be seen before clinical or echocardiographic manifestations are present.^30^ Cardiac uptake that is ≥ bone uptake (grade 2/3), or a heart-to-contralateral-lung uptake ratio ≥ 1.5 is considered a positive result for both wild-type and hereditary ATTR amyloidosis.

### Clinical case presentation 1

A 58-year-old man was referred to a cardiologist with heart failure symptoms (dyspnea New York Heart Association class II). He had no history of hospitalization for heart failure or prescribed diuretics. His brain natriuretic peptide (BNP) and N-terminal pro-BNP (NT-proBNP) levels were 345 and 865 pg/mL (normal: < 100 and < 300 pg/mL), respectively. His neurologic history included previous carpal tunnel surgery and progressive numbness in his feet over a 6-month period. The patient had abdominal discomfort and occasional diarrhea for 2 years (colonoscopy negative). Genetic assessment revealed a hereditary ATTR variant (p.V50M, formerly named V30M). The patient was referred to a cardiologist specializing in the management of cardiac amyloidosis. Additional investigations included echocardiogram, which revealed normal LV function, severe concentric LV hypertrophy, global longitudinal strain (GLS) –12% (normal: –16% to –22%) with preserved apical strain (“cherry-on-top” pattern), and no significant valve disease. A PYP nuclear scan showed a heart-to-contralateral-lung uptake ratio of 1.8 (normal: < 1.5) and grade 3 myocardial uptake (normal: grade 0 or 1). Small-fiber test results were abnormal, suggesting polyneuropathy.

### Clinical case discussion 1

This example documents the case of a patient with late-onset p.V50M ATTRv amyloidosis, which is more common in a nonendemic population such as that in Canada. Although the initial symptoms of heart failure were mild, co-occurring neurologic symptoms raised suspicion for ATTRv amyloidosis. In particular, gastrointestinal symptoms for 2 years and previous carpal tunnel surgery suggest amyloid has been accumulating for several years. Genetic testing revealed a p.V50M mutation in the *TTR* gene, which frequently presents as a mixed phenotype. This corresponds with the multisystem signs and symptoms present at initial consultation and is supported further by echocardiography and nuclear scan results, which confirmed cardiac involvement, as well as small-fiber tests that confirmed neurologic involvement.

## Clinical Question 2: How Should Patients with ATTRv Amyloidosis with Mixed Phenotype Be Treated?

### Background

Treatment for ATTRv amyloidosis should be initiated as soon as a patient meets eligibility requirements for disease-modifying therapy. Such requirements may include the following: a history of heart failure symptoms and New York Heart Association class I to III for ATTRv cardiomyopathy, or polyneuropathy disability stage I to ≤ IIIB or a familial amyloidotic polyneuropathy stage I or II with objectively assessed mild polyneuropathy. However, specific funding criteria may differ in each province. A swift diagnosis and early treatment offer the best chance at preserving quality of life and extending survival, particularly as current therapies aim to stabilize disease.^31^^,^^32^ Disease reversal is less common, particularly in those with advanced, longstanding disease.

There are 2 general approaches for the treatment of ATTRv amyloidosis: (i) stopping the formation of amyloid protein aggregates; and (ii) removing existing amyloid deposits. Currently available disease-modifying treatments for ATTRv amyloidosis focus on the first approach. These treatments include the following: (i) TTR stabilizers, which impede the dissociation of TTR tetramers into unstable monomers; and (ii) *TTR* gene silencers, which target TTR messenger RNA (mRNA) for degradation (Fig. 1). Table 2 outlines the phase-3 clinical trials leading to Health Canada approvals for ATTRv polyneuropathy and ATTR cardiomyopathy, as well as important ongoing phase-3 trials.

**Table 2:** Completed and ongoing phase-3 clinical trials in amyloid transthyretin variant (ATTRv) amyloidosis

Phase-3 trial	N	Study arms	Summary of results	Led to Health Canada indication?
**Polyneuropathy trials**				
NEURO-TTR^42^	172	Inotersen vs placebo	**Primary endpoint:** Inotersen was associated with a significant difference in change of mNIS+7 and QOL-DN scores from baseline at 66 wk vs placebo (*P* < 0.001, favouring inotersen) **Safety:** Serious events of glomerulonephritis and thrombocytopenia in inotersen arm (3% each)	Yes, but discontinued from market in Canada
NEURO-TTRansform^43^	168	Eplontersen vs historical placebo from NEURO-TTR (inotersen reference arm)	**Primary endpoint:** Eplontersen showed significantly greater reduction in serum TTR and more favourable change in mNIS+7 and QOL-DN scores at 66 wk (*P* < 0.001) **Safety:** Rates of thrombocytopenia and glomerulonephritis were similar in eplontersen and placebo arms	Yes
APOLLO-A^41^	225	Patisiran vs placebo	**Primary endpoint:** Patisiran was associated with a significant difference in change of mNIS+7 at 9 months vs placebo (*P* < 0.001, favouring patisiran) **Secondary endpoints:** Patisiran was associated with significantly better mNIS+7 QOL-DN scores, modified BMI, 10MWT, and R-ODS (*P* < 0.001) at 18 mo **Safety:** Infusion-related reactions occurred more frequently with patisiran	Yes
HELIOS-A^44^	164	Vutrisiran vs historical placebo from APOLLO-A (patisiran reference arm)	**Primary endpoint:** Vutrisiran was associated with a significant difference in change of mNIS+7 at 9 mo vs placebo (*P* < 0.001, favouring vutrisiran) **Secondary endpoints:** Vutrisiran was associated with significantly better QOL-DN scores, modified BMI, and gait speed (*P* < 0.001) **Safety:** Arthralgia was more frequent in the vutrisiran vs the placebo arm	Yes
**Cardiomyopathy trials (ATTRv or ATTRwt)**				
TTR protein stabilizers				
ATTR-ACT^33^	441	Tafamidis 80 mg and tafamidis 20 mg vs placebo	**Primary endpoint:** Tafamidis led to lower risk of death (HR 0.70, 95% CI 0.51–0.96) and cardiovascular complications (HR 0.70, 95% CI 0.56–0.81) **Secondary endpoints:** Tafamidis led to lower rate of decline on 6MWT and KCCQ-OS score (*P* < 0.001) at 30 mo **Safety:** Adverse events similar between groups	Yes
ATTRibute-CM ^36^	632	Acoramidis vs placebo	**Primary endpoint:** Hierarchical analysis of death (any cause), cardiovascular hospitalization, change in NT-proBNP, and change in 6MWT favoured acoramidis at 30 mo (*P* < 0.001) **Secondary endpoints:** Acoramidis led to lower decline on KCCQ-OS score (*P* < 0.001) **Safety:** Adverse events similar between groups	Not yet reviewed
*TTR* gene silencers				
APOLLO-B ^45^	360	Patisiran vs placebo	**Primary endpoint:** Patisiran led to a lower decline in change in 6MWT at 12 mo (*P* = 0.02) **Secondary endpoints:** Patisiran led to a lower decline in change in KCCQ-OS score (*P* = 0.04), but it was not significantly favoured in the composite end point of death (any cause), cardiovascular events, and change in 6MWT result **Safety:** Infusion-related reactions, arthralgia, and muscle spasms occurred more frequently with patisiran	No
HELIOS-B ^48^	655	Vutrisiran vs placebo	**Primary endpoint:** Vutrisiran led to lower risk of all-cause death and recurrent cardiovascular complications (HR 0.72; 95% CI 0.56–0.93) **Secondary endpoints:** Vutrisiran led to a lower risk of all-cause death (HR 0.65; 95% CI, 0.46–0.90) and lower decline in change in 6MWT result and KCCQ-OS score (*P* < 0.001) **Safety:** Adverse events similar between groups	Not yet reviewed
CARDIO-TTRansform (NCT04136171)	∼1400	Eplontersen vs placebo	Trial ongoing **Primary endpoint:** Composite outcome of cardiovascular mortality and recurrent cardiovascular events up to 140 wk	N/A
Amyloid depleters				
DepleTTR-CM (NCT06183931)	∼1000	ALXN2220 vs placebo	Trial ongoing **Primary endpoint:** Composite outcome of all-cause mortality and cardiovascular events (baseline up to 48 mo)	N/A
CRISPR-based gene therapy				
MAGNITUDE (NCT06128629)	∼765	NTLA-2001 vs placebo	Trial ongoing **Primary endpoint:** Composite outcome of cardiovascular mortality and cardiovascular events (baseline up to 18-48 mo)	N/A
MAGNITUDE-2 (NCT06672237)	∼50	NTLA-2001 vs placebo	Trial ongoing **Primary endpoint:** mNIS+7 (change from baseline at 18 mo), serum TTR (change from baseline to 29 d)	N/A
**Asymptomatic pathogenic TTR variant trials**				
ACT-EARLY (NCT06563895)	∼600	Acoramidis vs placebo	Trial ongoing **Primary endpoint:** Time to development of ATTR-CM and/or ATTR-PN	N/A

ATTR-CM, transthyretin amyloidosis with cardiomyopathy; ATTR-PN, ATTR with polynephropathy; ATTRwt, amyloid transthyretin wild-type; BMI, body mass index; CI, confidence interval; CRISPR, clustered regularly interspaced short palindromic repeat; HR, hazard ratio; N/A, not applicable; NT-proBNP, N-terminal pro B-type natriuretic peptide; KCCQ-OS, Kansas City Cardiomyopathy Questionnaire overall summary; mNIS+7, modified Neuropathy Impairment Score +7; QOL-DN; Norfolk Quality of Life-Diabetic Neuropathy; R-ODS, Rasch-built Overall Disability Scale; TTR, transthyretin; 6MWT, 6-minute walk test; 10MWT, 10-metre walk test.

Tafamidis is currently the only TTR stabilizer approved in Canada, and it is indicated for the treatment of adult patients with wild-type or variant ATTR cardiomyopathy. This indication is based on phase-3 clinical trial data showing reduced mortality and cardiovascular hospitalizations for patients with ATTR cardiomyopathy receiving tafamidis vs placebo (Table 2).^33^ Marginal benefits of tafamidis in patients with ATTRv polyneuropathy have been reported.^34^^,^^35^ As a result, this drug is approved for polyneuropathy in only some countries, not including Canada. Acoramidis, a potentially more potent stabilizer, recently has been shown to be beneficial in ATTR cardiomyopathy, but it is not yet available in Canada (Table 2).^36^ Of note, diflunisal was the first TTR stabilizer to demonstrate clinical benefit in ATTRv polyneuropathy^37^ and cardiomyopathy,^38^, ^39^, ^40^ but it is not approved by Health Canada for any ATTRv indication.

Four gene silencers have been approved in Canada for the treatment of ATTRv polyneuropathy based on more favourable changes in the modified neuropathy impairment score +7 (mNIS+7), compared to the scores of placebo controls (Table 2).^41^, ^42^, ^43^, ^44^ However, access to many of these agents is limited. Inotersen and eplontersen are antisense specific oligonucleotides that bind to complementary sequences on TTR mRNA and initiate its degradation through the nuclear RNase H1. Although inotersen was approved by Health Canada based on the NEURO-TTR trial, it has recently been discontinued from the market.^42^ Eplontersen is a next-generation antisense specific oligonucleotide with an N-acetyl galactosamine (GalNAc) tag to facilitate hepatic uptake and a longer dosing interval than inotersen (administered by an auto-injector monthly vs weekly). Eplontersen obtained Health Canada approval for use in treating ATTRv polyneuropathy based on the results from the NEURO-TTRansform study (Table 2).^43^

Patisiran is a small interfering RNA (siRNA) molecule that stimulates TTR mRNA degradation through the RNA-induced silencing complex (RISC). Vutrisiran, a next-generation siRNA, has structural adjustments that increase its stability and hepatic uptake, and it allows for subcutaneous administration every 3 months (vs intravenous delivery every 3 weeks for patisiran). Patisiran and vutrisiran are both indicated for the treatment of ATTRv polyneuropathy in Canada based on results from APOLLO-A (a phase-3, placebo-controlled trial of patisiran in patients with Familial Amyloid Polyneuropathy) and HELIOS-A (a phase 3, randomized trial of vutrisiran in patients with Familial Amyloid Polyneuropathy), respectively (Table 2).^41^^,^^44^

Although the initial phase-3 trials of *TTR* gene silencers were conducted in patients with ATTRv polyneuropathy and were not powered to assess the benefit in patients with cardiac symptoms, subgroup analyses of these studies in patients with markers suggestive of cardiomyopathy showed improvements in measures such as echocardiographic parameters, 6-minute walk test distances, and NT-proBNP levels.^45^, ^46^, ^47^

Studies evaluating *TTR* gene silencers specifically in ATTR cardiomyopathy have since been conducted (Table 2). The APOLLO-B trial study met its primary endpoint, showing a significantly reduced decline in the 6-minute walk test distance for those treated with patisiran vs placebo over 12 months.^45^ However, statistical significance was not achieved for other composite endpoints. The HELIOS-B study also met its primary endpoint, showing a reduced risk of death from any cause and recurrent cardiovascular events for vutrisiran vs placebo in the overall population of patients with ATTR cardiomyopathy.^48^

As TTR silencers currently are indicated in Canada only for ATTRv polyneuropathy, and TTR stabilizers are only indicated for ATTR cardiomyopathy, with limited evidence to support the use of these agents in combination, collaboration between cardiologists and neurologists is needed to determine the most appropriate therapy. A reasonable expectation is that medications demonstrating efficacy for both polyneuropathy and cardiomyopathy may become the standard of care for ATTRv amyloidosis. Currently, vutrisiran is the only agent with positive phase-3 trial data showing efficacy in both polyneuropathy and cardiomyopathy. Although vutrisiran may be preferred for treating mixed phenotype ATTRv amyloidosis at this time, particularly when both cardiac and neurologic involvement are similar in severity, this preference may evolve as additional data are reported. Eplontersen also may be considered in patients with mixed phenotype who have predominant neurologic symptoms, particularly as it has demonstrated efficacy in improving and/or stabilizing cardiac symptoms based on subgroup analyses of the NEURO-TTRansform study. Results from the phase-3 CARDIO-TTRansform trial are pending, which may confirm the efficacy of eplontersen use in patients with ATTRv cardiomyopathy, in addition to its currently proven efficacy in treating ATTRv polyneuropathy. As these gene silencers are not yet approved by Health Canada for patients with cardiomyopathy, patients with mixed phenotype ATTRv must qualify for these treatments based on the eligibility criteria for polyneuropathy. For patients with mixed phenotype ATTRv who have primarily cardiac symptoms with minimal neurologic involvement, use of tafamidis may be considered.

Therapies with novel mechanisms of action also are under investigation in ATTRv cardiomyopathy. These include CRISPR-Cas9 (for clustered regularly interspaced short palindromic repeats and CRISPR-associated protein 9) genome editing therapy, which aims to permanently knock down *TTR* gene transcription, as well as monoclonal antibodies that target misfolded TTR aggregates for macrophage-mediated phagocytosis (TTR depleters). Both novel therapeutic approaches have demonstrated efficacy in phase-1 studies and are being evaluated in the phase-3 MAGNITUDE, MAGNITUDE-2, and depleTTR-CM trials (Table 2).^49^^,^^50^

As the treatment landscape for mixed phenotype ATTRv amyloidosis evolves, factors such as drug access, availability, route and frequency of administration, and safety profile will play a larger role in treatment selection. These factors also are important to consider in the setting of intolerance, disease progression, or lack of response to first-line treatment.

### Clinical case presentation 2

A 54-year-old man with Afro-Caribbean ancestry was referred to a cardiologist to investigate a syncopal event. He reported dyspnea with exercise over the last 6 months but did not have heart failure hospitalizations and was not on diuretics. An ECG showed nonspecific T changes, and Holter investigations were normal. His NT-proBNP levels were 475 pg/mL (normal: < 300 pg/mL). A transthoracic echocardiogram showed concentric LV remodelling and grade 1 diastolic dysfunction. Interventricular septal wall and posterior wall thickness were mildly abnormal (13 mm and 13 mm, respectively; normal: < 12 mm). A GLS of –11% with apical sparing was noted (normal: –16% to –22%). The PYP nuclear scan was normal. Given the suggestive but nonspecific findings on echocardiography, cardiac MRI was ordered, which showed global moderate LV wall thickness, increased subendocardial late gadolinium enhancement, and increased extracellular volume fraction.

His neurologic history included recurrent numbness in the left hand and mild imbalance when walking. Autonomic symptoms included new-onset diarrhea with normal colonoscopy, abdominal fullness, and pharmaco-resistant erectile dysfunction for several years. Genetic testing revealed a *p.V142I TTR* mutation. The patient was referred to a neurologist for further examination. Results of the tilt-table test and the quantitative sudomotor axon reflex test (QSART) to investigate autonomic neuropathy were normal. Objective neurologic assessment was performed based on the Neuropathy Impairment Score (NIS). Results showed normal cranial nerves and segmental strengths and diminished deep tendon reflexes (left brachioradialis, quadriceps bilaterally and left Achilles, absent right Achilles). Sensory assessment found a slight decrease in sensitivity to pricks, from the fingertips to the middle of the hand, and significantly reduced light touch in the right big toe. The patient’s gait was fairly stable, but less assured than expected for his age. The patient qualified for treatment with a *TTR* gene silencer, and is doing better following initiation of patisiran, with some reversal of the neuropathic features. He has not developed symptomatic heart failure since his amyloidosis diagnosis.

### Clinical case discussion 2

This case illustrates the case of a patient with an initially subtle clinical presentation. Despite a negative PYP scan, genetic testing was pursued due to a high degree of suspicion for ATTRv amyloidosis based on the echocardiogram and MRI results, ancestry, and cardiac and neurologic symptom history. This patient was diagnosed with a *p.V142I TTR* variant, which occurs predominantly in patients of Afro-Caribbean descent and is historically associated with a predominantly cardiac phenotype; however, this case illustrates the heterogeneity in phenotypic presentation, with confirmation of neuropathy-related symptoms based on objective assessment.

After consultation between the cardiologist and neurologist, polyneuropathy was determined to be the dominant disease presentation, so a *TTR* gene silencer was prescribed to manage disease progression. Although the eligibility criteria for access to gene silencers via public funding vary by province, neurologic assessments were sufficient to qualify this patient for patisiran treatment in Quebec. Long-term analysis of gene silencer studies in ATTRv amyloidosis suggest that patients at an earlier disease stage may derive the most benefit from this therapy.^31^^,^^32^ In this case, the patient, who had mild symptoms, reported improvement in neuropathy symptoms and some reversal in neuropathic features. Exploratory analysis and prospective data from gene silencer studies also have suggested some benefit of these agents in stabilizing progression of ATTR-related cardiomyopathy. Although gene silencers currently are not indicated in Canada to treat ATTRv cardiomyopathy, they are a preferred option in patients with mixed phenotype, particularly vutrisiran, as mentioned above, although the HELIOS-B data were not available at the time of therapy selection for this patient.

## Clinical Question 3: How Should Patients with Mixed Phenotype ATTRv Amyloidosis Be Monitored Pre- and Post-Treatment?

### Background

Disease-modifying therapy is not yet indicated for asymptomatic patients with a known *TTR* mutation. However, careful monitoring of these patients is important to allow early identification of symptoms and therapy initiation, which may be more effective in preventing or slowing disease progression. Establishment of the predicted age of symptomatic disease onset (PADO) is needed to estimate the most appropriate time to initiate annual screening.^51^ The PADO is based on the specific *TTR* mutation involved and what has been historically reported as the typical age of symptomatic onset associated with that mutation, as well as the age of symptom onset for affected family members. Guidelines propose annual monitoring of presymptomatic *TTR* mutation carriers starting 10 years prior to the PADO.^51^ The ongoing phase-3 Acoramidis Transthyretin Amyloidosis Prevention Trial in the Young (ACT-EARLY) aims to determine whether early treatment with acoramidis can prevent or delay disease onset in asymptomatic *TTR* pathogenic variant carriers within 10 years of their PADO.^52^

A generally accepted protocol is that patients with symptomatic ATTRv amyloidosis receiving treatment be monitored by a cardiologist and neurologist every 6-12 months, at the discretion of the clinicians.^53^^,^^54^ Follow-up care typically includes assessments similar to those performed in diagnostic workup, as well as monitoring for changes in patient-reported outcomes. The ease and availability of the different assessments will play a role in determining the frequency with which they are performed.

The criteria for establishing disease progression and when a switch in therapy should be considered are not well defined. Given the rapid progression of untreated ATTRv amyloidosis, the rate of progression is an important consideration when deciding whether to continue or switch therapies. Management decisions for ATTRv amyloidosis should be made by an experienced multidisciplinary team. Expert consensus committees have proposed recommendations for defining progression of ATTR cardiomyopathy and polyneuropathy; however, evidence to suggest specific thresholds is limited, and many of the suggested measures are subject to intra- and inter-observer variability.^53^^,^^54^ For cardiomyopathy, the proposed thresholds for progression were based on changes in multiple domains, including clinical and functional measures of heart failure, serum markers, and imaging findings, such as increased LV wall thickness, decreased LVEF, and decreased GLS.^53^ A recent study by Ioannou and colleagues found that an NT-proBNP level increase of > 700 ng/L (absolute) and > 30% (relative), in combination with outpatient diuretic intensification, was significantly associated with an increased mortality risk for patients with ATTR cardiac amyloidosis, suggesting that these indicators could be a simple model used to measure disease progression.^55^

For polyneuropathy, the proposed definition of progression included either the worsening of several quantifiable symptoms, signs, or objective test results, the worsening of a symptom causing increased functional impairment, or the emergence of a new symptom.^54^ Recommended measures to assess for changes from baseline that could indicate progression included functional tests (eg, gait, polyneuropathy disability scale scores, grip strength), patient-reported outcomes, comprehensive neuromuscular examination (eg, NIS), and objective tests for somatic or autonomic neuropathy (eg, nerve conduction studies, and tests for small fiber and autonomic function if available; Fig. 2).^54^

### Clinical case presentation 3

A 33-year-old man was referred to a cardiologist to investigate symptoms of dyspnea. This patient had a family history of ATTRv amyloidosis (mother with severe polyneuropathy at age 50 years) and already was confirmed to be a carrier of the *TTR p.V50M* mutation. Echocardiography was performed, which showed structurally normal findings with normal strain. The ECG showed normal sinus rhythm with nonspecific T wave abnormalities. The patient did not have any prior hospitalizations or symptoms suggestive of heart failure and was not taking any cardiac medication, including diuretics. Due to his family history, a PYP scan was also conducted and classified as grade 3, suggesting amyloid deposition in the heart. All neuromuscular tests were normal, with no signs of neuropathy. Based on this presentation, the patient was not eligible for amyloidosis disease-modifying therapy. A yearly follow-up was suggested for assessment of changes on echocardiography (LV wall thickness, global longitudinal strain), ECG, and relevant biomarkers (eg, BNP, NT-proBNP). The patient remained stable, from a cardiac perspective, for 2 years; however, he developed tingling in the hands and feet. He was referred back to a neurologist and was found to have abnormal small-fiber test results. The patient was diagnosed with mixed phenotype ATTRv amyloidosis, and as neuropathy was the predominant phenotype, he was prescribed inotersen. This patient currently is being considered for treatment with vutrisiran due to inotersen no longer being available in Canada. The plan is for him to be monitored yearly by cardiology and every 6 months by neurology after initiation of treatment.

### Clinical case discussion 3

This case is that of a presymptomatic *TTR* mutation carrier who requires regular monitoring for the onset of symptomatic disease. Given the aggressive nature of amyloidosis related to the *TTR p.V50M* variant, and the relatively early onset of disease of the patient’s mother, initiation of regular monitoring is reasonable for this patient at age 33 years. Despite an initial presentation of dyspnea and evidence of amyloid deposition in the heart, echocardiogram results did not align with cardiac involvement, suggesting that the patient should continue to be monitored without initiation of treatment. Given that the PYP scan was already positive, but with no cardiac symptoms, a repeat scan was not deemed necessary in follow-up care. No guidelines or clear recommendations specify how nuclear scintigraphy should be used during monitoring. Some centres may repeat a PYP scan after 1 year on treatment, to assess for response. If the PYP scan is initially negative and a positive PYP scan will not change treatment (eg, as in case 2—the patient is mixed phenotype based on symptoms and echocardiogram and is receiving a gene silencer primarily to address polyneuropathy), an annual PYP scan is not necessary unless worsening heart failure symptoms or progression on echocardiography is observed. If a PYP scan is negative and the patient does not qualify to receive treatment for any indication, the recommendation is to repeat the PYP scan in a year.

Awareness of neurologic manifestations of *ATTR p.V50M* amyloidosis and careful examination by the cardiologist allowed for early identification of neuropathy symptoms and referral to a neurologist. As this patient had mixed phenotype ATTRv amyloidosis, a structured follow-up plan with both the cardiologist and neurologist was established. Given the family history and current neurologic predominance, a more frequent follow-up of once every 6 months with the neurologist was suggested.

## Conclusion

ATTRv amyloidosis remains a debilitating and life-threatening disease; however, earlier diagnosis and modern disease-modifying therapies have led to improved survival over the past decade.^10^ As disease presentation is heterogeneous and evolves over time, it is crucial that both cardiologists and neurologists are aware of signs and symptoms that should raise clinical suspicion of disease and that both specialists are involved in monitoring early on for new or worsening disease manifestations. This review provides a practical framework for the diagnosis, treatment, and monitoring of patients with mixed phenotype ATTRv amyloidosis from the perspective of both cardiologists and neurologists (Fig. 2). With novel therapeutic approaches on the horizon, management of ATTRv amyloidosis will continue to evolve, with the prospect of improving the quality of life and extending survival for these patients.
